# Combination of extramural venous invasion and lateral lymph node size detected with magnetic resonance imaging is a reliable biomarker for lateral lymph node metastasis in patients with rectal cancer

**DOI:** 10.1186/s12957-021-02464-3

**Published:** 2022-01-05

**Authors:** Tomoki Abe, Masayoshi Yasui, Hiroki Imamura, Chu Matsuda, Junichi Nishimura, Naotsugu Haraguchi, Nozomu Nakai, Hiroshi Wada, Hidenori Takahashi, Takeshi Omori, Hiroshi Miyata, Masayuki Ohue

**Affiliations:** Department of Gastroenterological Surgery, Osaka International Cancer Institute, Otemae 3-1-69, Chuo-ku, Osaka City, Osaka 541-8567 Japan

**Keywords:** Rectal cancer, Lateral lymph node, Lateral lymph node dissection, Extramural venous invasion, Magnetic resonance imaging

## Abstract

**Purpose:**

Pathological extramural venous invasion (EMVI) is defined as the active invasion of malignant cells into veins beyond the muscularis propria in colorectal cancer. It is associated with poor prognosis and increases the risk of disease recurrence. Specific findings on MRI (termed MRI-EMVI) are reportedly associated with pathological EMVI. In this study, we aimed to identify risk factors for lateral lymph node (LLN) metastasis related to rectal cancer and to evaluate whether MRI-EMVI could be a new and useful imaging biomarker to help LLN metastasis diagnosis besides LLN size.

**Methods:**

We investigated 67 patients who underwent rectal resection and LLN dissection for rectal cancer. We evaluated MRI-EMVI grading score and examined the relationship between MRI-EMVI and LLN metastasis.

**Results:**

Pathological LLN metastasis was detected in 18 cases (26.9%), and MRI-EMVI was observed in 32 cases (47.8%). Patients were divided into two cohorts, according to LLN metastasis. Multivariate analyses demonstrated that higher risk of LLN metastasis was significantly associated with MRI-EMVI (*P* = 0.0112) and a short lateral lymph node axis (≥ 5 mm) (*P* = 0.0002). The positive likelihood ratios of MRI-EMVI alone, LLN size alone, and the combination of both factors were 2.12, 4.84, and 16.33, respectively. Patients negative for both showed better 2-year relapse-free survival compared to other patients (84.4% vs. 62.1%, *P* = 0.0374).

**Conclusions:**

MRI-EMVI was a useful imaging biomarker for identifying LLN metastasis in patients with rectal cancer. The combination of MRI-EMVI and LLN size can improve diagnostic accuracy.

**Supplementary Information:**

The online version contains supplementary material available at 10.1186/s12957-021-02464-3.

## Introduction

Pathological extramural venous invasion (EMVI) is defined as the active invasion of malignant cells into veins beyond the muscularis propria in colorectal cancer [1]. Pathological EMVI occurs in about one-third of patients with rectal cancer and is associated with higher rates of local and distant recurrence and poor prognosis [2–6]. Evaluation of pathological EMVI requires a surgical specimen; therefore, EMVI-based predictions of recurrent risk and prognosis are only possible after surgery.

Smith et al. [7] used magnetic resonance imaging (MRI) to examine specific features of the rectum and mesorectum. They identified features associated with pathological EMVI, which they termed “MRI-EMVI.” These features included signal intensity within vessels, obvious irregular vessel contour, and nodular vessel expansion by definite tumor signal. Many recent reports substantiate the clinical significance of MRI-EMVI. Findings of MRI-EMVI are associated with T stage, mesorectal lymph node metastasis, and a positive circumferential resection margin (CRM) [8]. Furthermore, MRI-EMVI exhibits strong associations with distant recurrence, prognosis, and pathological EMVI [9–13]. These results support that investigating MRI-EMVI could be useful in making treatment decisions. In fact, the rectal cancer guidelines established by the European Society for Medical Oncology (ESMO) [14] state that MRI-EMVI is a significant risk factor for local recurrence, and recommend assessing the MRI-EMVI before treatment.

In recent years, the clinical relevance of LLN for rectal cancer is garnering attention [15, 16]. It is important to consider the appropriate treatment of suspicious LLNs. The size of LLN is the most common parameter in assessments of LLN metastasis [17]. Previous reports also showed that an irregular border or mixed-signal intensity of LLN on MRI findings was useful for the diagnosis of LLN metastasis [18, 19]. However, the diagnostic ability is limited by any method.

In the present study, we aimed to determine whether MRI-EMVI was correlated with LLN metastasis in patients with rectal cancer. We also investigated better methods for the detection of LLN metastasis.

## Materials and methods

### Patients

**Fig. 1:**
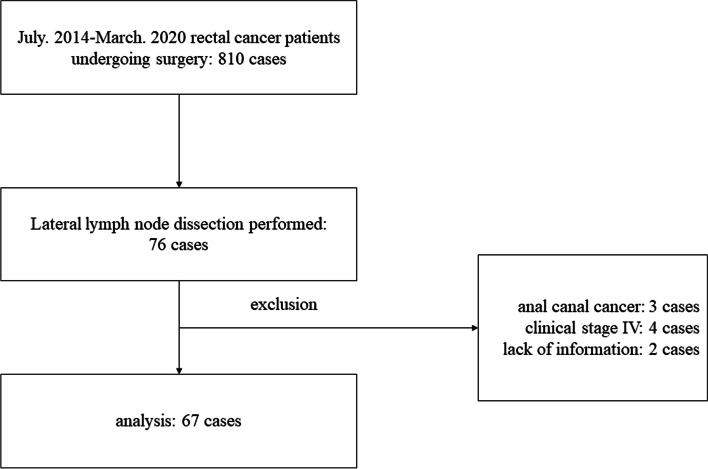
Flowchart of participants of the study

For this study, we investigated 810 patients who underwent rectal resection due to rectal cancer between July 2014 and March 2020 at our institute. Patients underwent pelvic MRI imaging, followed by rectal resection and LLN dissection, for rectal cancer involving a tumor with the lower border located distal to the peritoneal reflection. Patients were diagnosed with rectal cancer based on biopsy analysis prior to surgery. At our institute, surgery was recommended according to contemporary guidelines established by the Japanese Society for Cancer of the Colon and Rectum (guidelines published in 2014, 2016, and 2019, for the treatment of colorectal cancer) [20–22]. LLN dissection was performed in 76 patients. Patients with distant metastases (clinical stage IV) had undergone not complete LLN dissection, but LLN pickup. Patients with anal canal cancer and missing records were excluded. Finally, a total of 67 patients from our database were eligible for study inclusion (Fig. 1).

### Data collection

For all included patients, we collected clinicopathological data from a prospectively maintained database, including patient characteristics, laboratory investigations, and pathological staging. We also collected and evaluated information from MRI investigations.

### Histopathology

Histopathological assessments were performed to determine the T stage, N stage, presence of vascular invasion, and histopathological involvement within the CRM. A pathological CRM was defined as the presence of tumor tissue located ≤ 1 mm from the resection plane [23].

### MRI acquisition

We analyzed all pre-surgical plain MRI scans, which were obtained using a 1.5 Tesla MRI unit (SIEMENS Healthineers, MAGNETON Aera) or a 3.0 Tesla MRI unit (SIEMENS Healthineers, MAGNETON Skyra or MAGNETON Prisma). The standard imaging protocol included a sagittal T2-weighted (T2W) fast spin echo, and an oblique axial thin-section T2W (TR: 4000, TE: 104; slice: 4 mm; matrix: 320 × 326; FOV: 220 × 220; plane resolution: 0.688 mm).

### Quantitative assessment of MRI images

In MRI data assessments, we determined the T stage, N stage, anal verge on the MRI (MRI-AV), length of the LLN short axis, mesorectal fascia (MRF) involvement, and the MRI-EMVI. We evaluated images obtained by MRI at the time of diagnosis, with or without preoperative therapy. These parameters were analyzed independently by two investigators (TA, HI) who were blinded to patient clinical information. They received instruction from radiologists regarding the assessment of MRI.

**Fig. 2:**
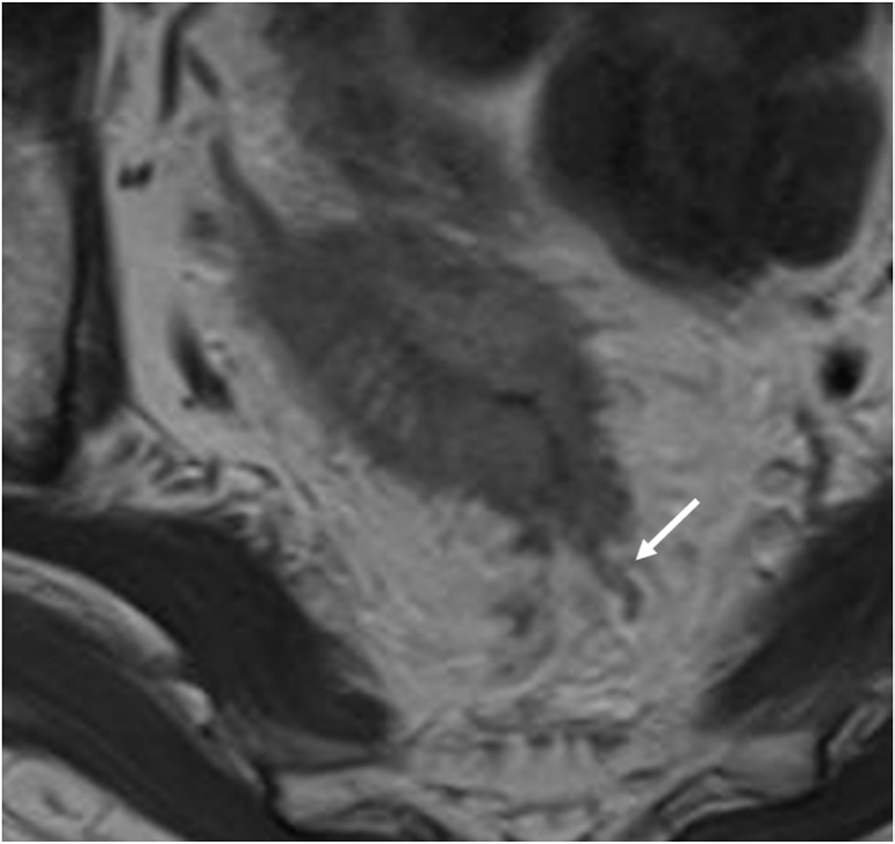
Extramural venous invasion detected with magnetic resonance imaging. Tumor signal intensity spread beyond the rectal wall. And irregular vessel contour or nodular expansion of vessel with definite tumor signal is demonstrated. MRI-EMVI score 4 (white arrow)

To grade the MRI-EMVIs, we adopted the scoring system proposed by Smith et al. [7]. Grades 0–2 were defined as negative for MRI-EMVI (no detectable disease), while grades 3 and 4 were defined as positive for MRI-EMVI (detectable disease). A grade 4 MRI-EMVI appeared with an obvious irregular vessel contour, or nodular vessel expansion by definite tumor signal (Fig. 2). When an evaluation was inconsistent, the evaluators discussed differences to reach a consensus on the MRI-EMVI score. MRF involvement was defined as positive if the tumor edge was ≤ 1 mm from the mesorectal fascia.

### Treatment and surgery

The treatment strategies for individual patients were determined by multidisciplinary cancer teams. Lower rectal cancer was defined as a tumor with the lower border located distal to the peritoneal reflection. Preoperative therapy (neoadjuvant chemotherapy [NAC] and neoadjuvant chemoradiotherapy [CRT]) was administered in cases of advanced cancer with suspected invasion to the levator muscle. Indications for LLND were either lower rectal cancer with cT3–4 or T1–2 rectal cancer with suspected metastasis to LLN. All patients underwent total mesorectal resection (TME) or partial mesorectal excision (ME), followed by LLN dissection or lymphadenectomy in the internal pudendal artery area, internal iliac artery area, and obturator region.

At our institute, the NAC regimen was capecitabine/oxaliplatin (CapeOX). The CapeOX regimen consisted of 130 mg/m^2^ of oxaliplatin provided as an intravenous infusion on day 1 of each 3-week cycle. Capecitabine (1000 mg/m^2^) was taken orally twice daily, from after dinner on day 1 to after breakfast on day 15, followed by a 7-day rest. NAC was administered for a total of 12 weeks. Neoadjuvant CRT consisted of a total dose of 50.4 Gy and oral capecitabine (same as NAC).

### Statistical analysis

The data are expressed as the mean ± standard deviation. Categorical variables were compared using the chi-square test, and continuous variables using Student’s *t* test. Any variable deemed significant (*P* < 0.05) in the univariate analysis was a candidate for the multivariate analysis. Multivariate analyses were performed with logistic regression. The optimal cut-off values for continuous variables were determined with receiver operating characteristics (ROC) analysis, when necessary. All analyses were conducted using JMP 13 software (SAS Institute, Cary, NC, USA). A *P* value of < 0.05 was considered to indicate statistical significance.

### Ethical guidelines

This study was approved by the Human Ethics Review Committee of the Osaka International Cancer Institute (no. 18033).

## Results

### Associations with LLN metastases

Our analysis included a total of 60 patients from our database, including 38 males and 29 females, with a median age of 65 years (range: 33–83 years). Preoperative therapy was performed in 15 cases (23.4%). MRI-EMVI was detected in 32 cases (47.8%), and 18 cases (26.9%) were pathological LLN positive. We calculated the kappa statistic to assess the reproducibility for MRI-EMVI of two researchers. The kappa statistic is 0.7312. Table 1 summarizes the patients’ clinical characteristics. Patients were divided into two groups, based on whether LLN metastasis was present (LLN-positive group) or absent (LLN-negative group). Patient factors did not significantly differ between these groups.

**Table 1:** Univariate analysis results show factors associated with pathological lateral lymph node metastasis

Factors	LLN-positive (***n*** = 18)	LLN-negative (***n*** = 49)	Univariate analysis (***P*** value)
**Patient factors**			
Age (years)	66.5 (47-83)	65 (33-78)	0.2529
Gender (male/female)	9/9	29/20	0.5024
Preoperative therapy (none/chemotherapy/chemoradiotherapy)	13/2/3	39/6/4	0.6262
cT (2/3/4a/4b)	0/8/2/8	3/34/4/8	0.0641
Clinically suspected mesorectal LN metastasis (+/−)	16/2	25/24	**0.0027**
**MRI factors**			
MRI-AV (mm)	34.5 (9-66)	36 (0.5-95)	0.4613
Enlarged LLN (positive/negative) ^a^	16/2	9/40	**< 0.0001**
MRI-EMVI (positive/negative)	14/4	18/31	**0.0024**
MRF involvement (positive/negative)	9/7	17/29	0.1800
**Surgical factors**			
Approach to LLN dissection (laparoscopy/open)	5/13	32/17	0.0058
Operation (LAR/sLAR/ISR/APR/TPE)	1/4/1/10/2	7/27/1/13/1	0.0500
**Pathological factors**			
Tumor size (mm)	35 (30–70)	50 (30–80)	0.6715
pT (≤ 2/≤ 3)	18/0	41/8	0.0289
Mesorectal LN metastasis (positive/negative)	12/6	22/27	0.1114
v (positive/negative)	16/2	37/12	0.2097
ly (positive/negative)	5/13	6/43	0.1443
Perineural invasion (positive/negative)	8/10	24/23	0.6326
Differentiation (Well or moderate/poor)	15/3	47/2	0.1044
CRM (0/1)	18/0	49/0	–
**Postoperative course**			
Recurrence (positive/negative)	5/13	13/36	0.9189
Site of overall recurrence (local recurrence/distant recurrence/local and distant recurrence)	0/4/1	2/10/1	0.4177
LLN recurrence (yes/no)	1/4	1/12	0.4782

Values are the median (range) or number, as indicated

*LN* lymph node, *MRI* magnetic resonance imaging, *AV* anal verge, *LLN* lateral lymph node, *EMVI* extramural venous invasion, *MRF* mesorectal fascia, *LAR* low anterior rectectomy, *sLAR* super low anterior rectectomy, *ISR* intersphincteric resection, *APR* abdominoperineal resection, *TPE* total pelvic exenteration, *v* venous invasion, *ly* lymphatic invasion, *CRM* circumferential resection margin

^a^Positive/negative value of the short axis of lateral lymph node (mm) (≥ 5/< 5)

**Fig. 3:**
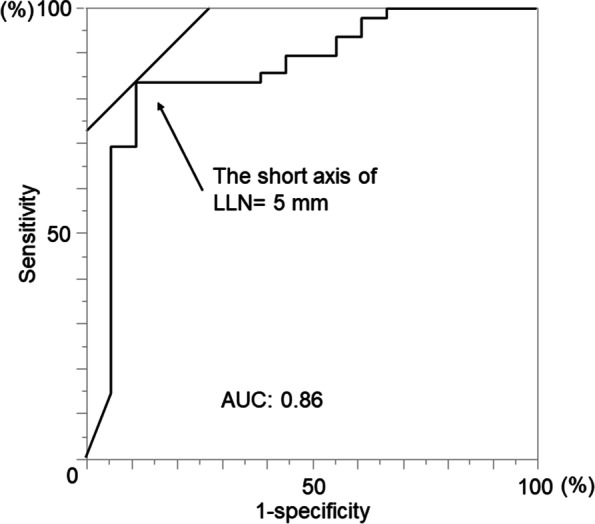
ROC analysis of the short axis of LLN for the risk of LLN metastasis. ROC analysis of the short axis of LLN is depicted. The cut-off value of the short axis was 5 mm, which yielded a sensitivity of 89% and a specificity of 82%. The value of AUC for the risk of malignancy was 0.86

ROC analysis of the LLN short axis showed a preferable AUC of 0.86, with a cut-off value at 5 mm (Fig. 3). Univariate analysis identified three parameters that significantly differed between the LLN-positive group vs. the LLN-negative group: enlarged LLN (≥ 5 mm) (88.9% vs. 18.4%, *P* < 0.0001), incidence of MRI-EMVI (77.8% vs. 36.7%, *P* = 0.0024), and the rate of clinically suspected mesorectal lymph node metastasis (88.9% vs. 51.0%, *P* = 0.027). An additional factor tended to be higher in the LLN-positive group vs. the LLN-negative group: the proportion of patients with clinical stage T3 or higher (100% vs. 93.9%, *P* = 0.064). The two groups did not differ in the incidence of lymphovascular invasion, the recurrence rate, or the site of recurrence.

### Multivariate analysis for LLN metastasis

**Fig. 4:**
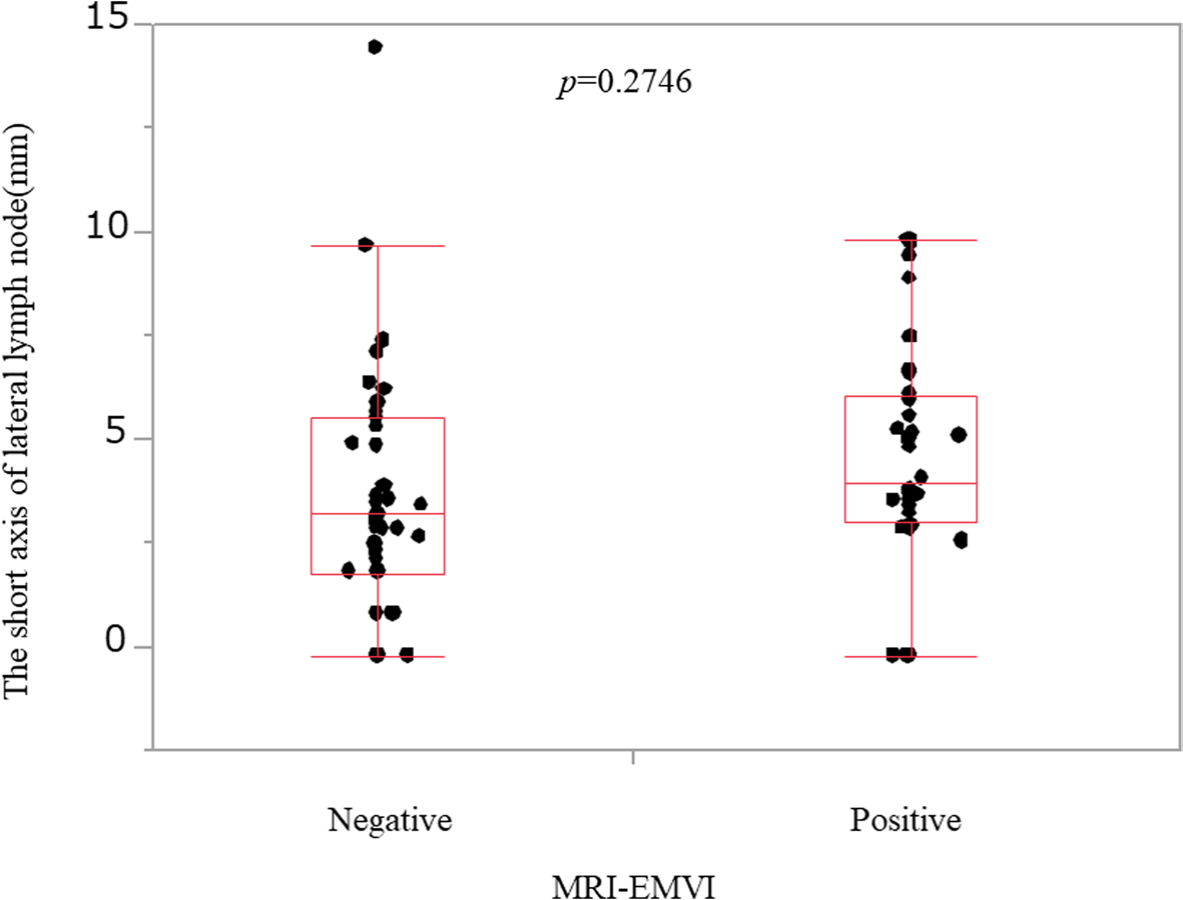
Correlation between MRI-EMVI and the short axis of lateral lymph node. There was no correlation between MRI-EMVI grade and the short axis of LLN

The four factors (MRI-EMVI, enlarged LLN, clinically suspected mesorectal lymph node metastasis, and clinical T) were included in a multivariate analysis. The results revealed that MRI-EMVI and enlarged LLN were independent risk factors for LLN metastasis: MRI-EMVI (*P* = 0.0112, OR: 12.28, 95% CI: 1.77–85.13) and enlarged LLN (*P* = 0.0002, OR: 50.14, 95% CI: 6.61–380.38) (Table 2). We found no correlation between MRI-EMVI grade and the LLN short axis (*P* = 0.2746) (Fig. 4).

**Table 2:** Multivariate analysis result show associations with LLN metastasis

Factor	***P*** value	Odds ratio	95% CI
MRI-EMVI (positive/negative)	**0.0112**	12.275	1.7700–85.1264
Enlarged LLN^a^ (positive/negative)	**0.0002**	50.137	6.608–380.3766
cT (≤ 2/≤ 3)	0.9938	526420.31	–
clinically suspected mesorectal LN metastasis (+/−)	0.1566	4.7811	0.5487–41.6596

*MRI* magnetic resonance imaging, *EMVI* extramural venous invasion, *LLN* lateral lymph node, *cT* clinical T, *LN* lymph node, *CI* confidence interval

^a^Positive/negative value of the short axis of lateral lymph node (mm) (≥ 5/< 5)

### Diagnostic ability by combination of MRI-EMVI and LLN size

Various cut-off values of LLN size were set in order to set the optimum LLN size in combination with MRI-EMVI. The positive predictive value (PPV), negative predictive value (NPV), and positive likelihood ratio (PLR) for LLN metastasis in cases with both positive MRI-EMVI and enlarged LLN were calculated. When the cut-off value was 5 mm, PPV and NPV and PLR were 0.86, 0.89, and 16.33, respectively. When the cut-off value was 6 mm, PPV and NPV and PLR were 0.78, 0.81, and 9.53, respectively. When the cut-off value was 7 mm, PPV and NPV and PLR were 0.80, 0.77, and 10.89, respectively. The PPV, NPV, and PLR were the highest when the cut-off value of LLN size was set at 5 mm. Table 3 shows the PPV, NPV, and PLR for MRI-EMVI, LLN size, and both two indexes positive, in association with LLN metastasis. In cases positive with both positive MRI-EMVI and enlarged LLN, the positive likelihood ratio for LLN metastasis was 16.33.

**Table 3:** Positive and negative predictive values and likelihood ratio for the two factors independently associated with LLN metastasis

Factor	Sensitivity	Specificity	PPV	NPV	Accuracy	PLR	NLR
MRI-EMVI (positive/negative)	0.78	0.63	0.44	0.89	0.67	2.12	2.85
Enlarged LLN (positive/negative)^a^	0.89	0.82	0.64	0.95	0.84	4.84	7.35
Positive MRI-EMVI and enlarged LLN /the others^b^	0.67	0.96	0.86	0.89	0.88	16.33	2.88
Negative MRI-EMVI and no enlarged LLN/the others^c^	1	0.49	0.42	1	0.63	1.96	–

*MRI* magnetic resonance imaging, *EMVI* extramural venous invasion, *LLN* lateral lymph node, *PPV* positive predictive value, *NPV* negative predictive value, *PLR* positive likelihood ratio, *NLR* negative likelihood ratio

^a^Positive/Negative value of the short axis of lateral lymph node (mm) (≥ 5/< 5)

^b^positive MRI-EMVI and no enlarged LLN, negative MRI-EMVI and enlarged LLN, or negative MRI-EMVI and no enlarged LLN

^c^positive MRI-EMVI and no enlarged LLN, negative MRI-EMVI and enlarged LLN, or negative MRI-EMVI and no enlarged LLN

### Patients negative for both MRI-EMVI and enlarged LLN had significantly good relapse-free survival

Kaplan-Meier curves were generated to compare relapse-free survival (RFS) between patients who were negative for both MRI-EMVI and enlarged LLN versus all other patients. The median follow-up period was 22 months (range: 4–69 months). Patients who were negative for both MRI-EMVI and enlarged LLN exhibited 1- and 2-year RFS rates of 91.5% and 84.4%, respectively. All other patients exhibited 1- and 2-year RFS rates of 74.5% and 62.1%, respectively. Significant differences were seen between these groups (*P* = 0.0374) (Supplemental Fig. 1).

## Discussion

Suppressing local recurrence is among the most important issues in treating rectal cancer. The most common site of local recurrence is the LLN [24], and LLN recurrence reduces quality of life due to edema and pain, etc. Identifying patients at high risk of LLN recurrence will help decide on the most appropriate treatment for controlling local recurrence. Enlarged LLN is reportedly a predictor of LLN metastasis [24, 25]. In our present study, we demonstrated that MRI-EMVI and enlarged LLN were each independently associated with LLN metastasis. Moreover, the combination of these two factors could more accurately predict pathological LLN metastasis. Normally, when the cutoff value of LN size is smaller, sensitivity is increased and PPV is decreased. However, it was found that both PPV and PLR could be maintained high in any LN size when combined with MRI-EMVI.

While mesorectal excision is the standard surgical procedure for rectal cancer, various treatment strategies have been developed for advanced rectal cancer [26–28]. In East Asia, including Japan, advanced lower rectal cancer is generally treated by ME plus LLN dissection. A previous Japanese retrospective multicenter study reported an 18.1% rate of LLN metastasis among patients with T3–4 lower rectal cancer [27]. In East Asia, the lateral lymph nodes are considered to be the regional lymph nodes [29]. Therefore, according to the 2019 guidelines established by the Japanese Society for Cancer of the Colon and Rectum, LLN dissection is required when a tumor invades beyond the muscularis propria (T2), and the lower tumor border is distal to the peritoneal reflection. In our present study, LLN dissection was performed in all applicable cases according to Japanese guidelines, enabling us to investigate the relationships between preoperative MRI-EMVI, LLN size, and pathological LLN metastasis. On the other hand, in Western countries, LLN metastasis is considered a distant disease and is thus treated with preoperative therapy rather than LLN dissection, and the standard strategy for lower rectal cancer is CRT followed by ME. Anania et al. [30] analyzed 34 articles and 29 studies regarding the role of LLN dissection during TME. They suggested that TME with LLN dissection does not offer an oncological advantage over TME without LLN dissection after neoadjuvant CRT. In addition, there is an option of watch and wait strategy for cases with clinical complete response after neoadjuvant treatment [31, 32]. Despite the different treatment strategies between Japan and Western countries, the local recurrence rates are similar [30, 33]. Each treatment strategy has its advantages and disadvantages, and therefore, the treatment strategy is still controversial.

The ESMO rectal cancer guidelines [14] recommend assessing for MRI-EMVI before treatment. MRI-EMVI has been shown to predict the risks of both local recurrence and synchronous/metachronous metastases. In cases positive for MRI-EMVI, a short course of preoperative radiotherapy (SCPRT) or CRT is recommended, with the aim of reducing recurrence. Previous studies reveal correlations between MRI-EMVI and prognosis in terms of overall survival, RFS, and increased distant recurrence [7, 9, 12, 34, 35]. Smith et al. [7] found significantly a worse 3-year RFS rate among patients positive for MRI-EMVI (35.0%) compared to patients negative for MRI-EMVI (74.1%). Similarly, van den Broek et al. [35] showed worse overall survival among patients with MRI-EMVI compared to patients without MRI-EMVI. Moreover, a meta-analysis [9] revealed that synchronous and metachronous metastases were more frequent among patients positive for MRI-EMVI compared to patients negative for MRI-EMVI.

While some previous studies have reported an association between MRI-EMVI and local recurrence [10, 35, 36], others have not identified this association. Schaap et al. [36] showed that MRI-EMVI was related to general local recurrence; however, this association was no longer observed when the analysis was limited to lateral and presacral lesions. Additionally, van den Broek et al. [35] reported that MRI-EMVI was not associated with local recurrence rates. However, these two studies were based on MRI-EMVI evaluation prior to preoperative treatment; therefore, the results might have reflected that preoperative treatment suppressed local recurrence. Similarly, in our present study, we found that local recurrence rates did not significantly differ between MRI-EMVI-positive and MRI-EMVI-negative cases. Since LLN dissection was performed in all cases, we speculate that LLN dissection might have suppressed local recurrence, particularly LLN recurrence.

The JCOG0212 trial [37] was conducted to confirm the noninferiority of ME alone compared to ME + LLN dissection, with relapse-free survival as the primary end-point. Their intent-to-treat analysis results did not demonstrate the noninferiority of ME alone compared to ME + LLN dissection. Additionally, the proportion of local recurrences was lower in the ME + LLN dissection group compared to in the ME alone group (7.4% vs. 12.6%, *P* = 0.024). The JCOG0212 trial targeted patients without LLN enlargement; however, even in these cases, local recurrence was possible even if LLN dissection was performed. In our present study, only two patients experienced LLN recurrences. These patients underwent LLN dissection, but did not receive preoperative therapy, and both were positive for MRI-EMVI. In these cases, the ME and LLN dissection were inadequate for disease control. As suggested by the ESMO, when a patient is positive for MRI-EMVI at baseline, it might be beneficial to perform preoperative treatment to suppress micrometastases in the LLN. On the other hand, even with the performance of both neoadjuvant chemoradiotherapy (NACRT) and ME, metastasis to the LLN remains a significant clinical concern [38]. Haanappel et al. [39] demonstrated that compared to patients without LLN metastases, patients with clinically suspected LLN metastases exhibited a clinically relevant trend toward worse 5-year local recurrence-free and disease-free survival. They suggested that in cases with clinically suspected LLN metastases, NACRT alone might not be sufficient treatment and that the addition of LLN dissection should be considered.

Ogura et al. [24] reported that among patients with enlarged LLNs (short axis ≥ 7 mm), the 5-year LLN local recurrence rate was lower with (C)RT plus ME plus LLN dissection (5.7 %) compared to with (C)RT plus ME (19.5%). As mentioned above, some studies suggest that patients should be properly evaluated for LLN metastasis and that those with LLN metastasis should undergo LLN dissection as well as preoperative treatment. In patients with strongly suspected LLN metastasis, multidisciplinary therapeutic interventions (e.g., preoperative therapy followed by ME and LLN dissection) might suppress LLN recurrence. Hamabe et al. [40] reported that stratification based on MRI-EMVI and LLN size could be used to assess individual risk of LLN metastasis. In our present study, pretreatment MRI-EMVI and LLN size were associated with LLN metastasis. Patients who were negative for both MRI-EMVI and enlarged LLN showed a better RFS rate. On the other hand, patients who were positive for MRI-EMVI and/or enlarged LLN exhibited a high risk of local and distant recurrence. Thus, in such cases, we may consider applying total neoadjuvant therapy (TNT), which is currently considered the most promising technique. Notably, Ogura et al. [24] reported that LLN dissection had the advantage of suppressing local recurrence. However, performing both pretreatment TNT and LLN dissection may increase technical difficulties, postoperative complications, and dysfunction. It is presently controversial whether LLN dissection should be performed in cases treated with NACRT or TNT. Therefore, an accurate diagnostic method is required. In patients positive for both MRI-EMVI and enlarged LLN, PPR for pathological LLN metastasis was 16.3, which is extremely informative and may help in treatment selection. To our knowledge, this study was the first to evaluate the positive likelihood ratio of MRI-EMVI for the purpose of identifying cases at a high risk of pathological LLN metastasis.

As mentioned earlier in this section, Anania et al. [30] reported that TME with LLN dissection does not offer an oncological advantage over TME without LLN dissection after neoadjuvant CRT. They suggested that there is no place for ‘routine’ LLN dissection in the management of rectal cancer. In other words, it is important to perform “selective” LLN dissection, and establishing a selection method is an important issue. Ogura et al. [41] reported that lymph node size after preoperative treatment may be useful in determining the indication of LLND. To establish the indication of selective LLN dissection, we are planning to analyze the usefulness of MRI-EMVI after neoadjuvant treatment to predict LLN metastasis only for preoperatively treated cases.

This study had some limitations. First, the patient selection for preoperative treatment was biased. Preoperative treatments, such as CRT, can affect pathological lateral lymph node metastasis. However, preoperative treatment cannot be a confounder in our study, because we evaluated the status of pathological LLN metastasis and MRI-EMVI grade before preoperative treatment rather than after preoperative treatment. Second, in Japan, after completion of rectal resection, we divided the resected specimen into the intestine and the mesentery before performing pathological examinations. We collect visible lymph nodes from the mesentery one by one. Since pathological EMVI is defined as the active invasion of malignant cells into veins beyond the muscularis propria, it is difficult to evaluate by Japanese-fashioned pathological examination. Although pathological concordance cannot be demonstrated, we believe that MRI-EMVI plays an important and unique role as a preoperative clinical characteristic for diagnosing LLN.

In conclusion, our present results revealed that MRI-EMVI could be a useful imaging biomarker for LLN metastasis of rectal cancer. Moreover, the combination of MRI-EMVI and LLN size could improve diagnostic accuracy and identify cases at a high risk of local recurrence. High-risk cases of rectal cancer may require multidisciplinary therapeutic interventions.

## Supplementary Information

**Additional file 1: Supplemental Figure 1.** Relapse-free survival rate in patients with ‘both negative MRI-EMVI and no enlarged LLN’. and the other patients.

## Data Availability

The data is included in this manuscript as supplementary material.
