# Supplementary figures and images for: Combination of extramural venous invasion and lateral lymph node size detected with magnetic resonance imaging is a reliable biomarker for lateral lymph node metastasis in patients with rectal cancer
Source: World J Surg Oncol. 2022 Jan 5;20:5. doi: 10.1186/s12957-021-02464-3 (PMC8728915; doi:10.1186/s12957-021-02464-3)

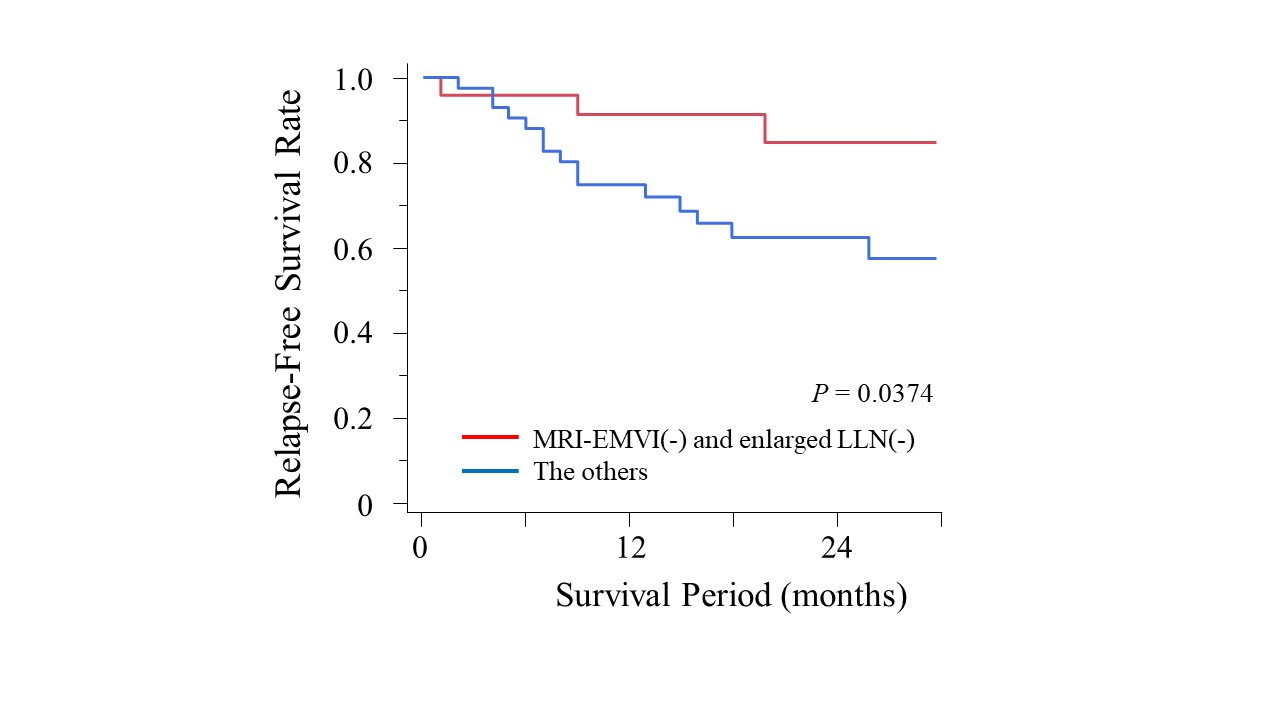

Supplement: Supplementary file 1 — Additional file 1: Supplemental Figure 1. Relapse-free survival rate in patients with ‘both negative MRI-EMVI and no enlarged LLN’. and the other patients. [file 12957_2021_2464_MOESM1_ESM.tif]
